# *Amphimas pterocarpoides* harms.: An Evaluation of flavonoid and phenolic contents, wound healing, anthelmintic and antioxidant activities of the leaves and stem bark

**DOI:** 10.1016/j.heliyon.2021.e08261

**Published:** 2021-10-27

**Authors:** Evelyn Asante-Kwatia, Silas Adjei, Yakubu Jibira, Lord Gyimah, George Adjei-Hinneh, Isaac Kingsley Amponsah, Abraham Yeboah Mensah

**Affiliations:** a Department of Pharmacognosy, Faculty of Pharmacy and Pharmaceutical Sciences, College of Health Sciences, Kwame Nkrumah University of Science and Technology, Kumasi, Ghana; b Department of Herbal Medicine, Faculty of Pharmacy and Pharmaceutical Sciences, College of Health Sciences, Kwame Nkrumah University of Science and Technology, Kumasi, Ghana; c Department of Pharmacology, Faculty of Pharmacy and Pharmaceutical Sciences, College of Health Sciences, Kwame Nkrumah University of Science and Technology, Kumasi, Ghana; d Department of Pharmaceutical Sciences, Sunyani Technical University, Sunyani, Ghana

**Keywords:** *Amphimas pterocarpoides*, Dermal excision, Worms, Oxidative stress, HPLC

## Abstract

The present study evaluated the wound healing, anthelmintic and antioxidant potentials of crude methanol extracts and fractions (petroleum ether, ethyl acetate and methanol) of the leaves and stem bark of *Amphimas pterocarpoides*. Wound healing activity was determined by the dermal excision model in rats; anthelmintic activity was evaluated by the adult worm motility test using the adult Indian worm, *Pheretima postuma*. Total flavonoid, phenolic content and antioxidant activity were assessed by the aluminum chloride colorimetric, Folin Ciocalteu, 1, 1-diphenyl-2-picrylhydrazyl (DPPH) free radical scavenging and total antioxidant capacity (TAC) assays respectively. HPLC/UV fingerprints were developed for quality control. The maximum amount of phenolics and flavonoids were detected in the methanol fractions of the stem bark (225.0 ± 20.0 mg/g gallic acid equivalent (GAE) and 201.0 ± 1.41 mg/g quercetin equivalent (QCE) respectively) and leaves (84.54 ± 1.36 mg/g GAE and 130.7 ± 1.71 mg/g QCE, respectively). Both leaf and bark displayed remarkable free radical scavenging and TAC with the highest effect given by the methanol fractions. Significant (*p* < 0.05) wound contraction was achieved by topical application of the leaf (APL) and stem bark (APS) ointments (5–15%) with >90 % wound surface closure for 1% silver sulphadiazine, APS 15% and APL 10% treated groups by day 15. APL and APS demonstrated a concentration- and time-dependent paralysis and mortality of the *P. posthuma* with APL (6.25 mg/mL) causing worm paralysis at 82.60 min and death at 93 min, better than 10 mg/mL albendazole (paralysis at 76.30 min; death at 117 min). Tannins, triterpenoids, phytosterols, flavonoids, saponins and coumarins were detected in the leaves and bark. The results have proven the potential of *A. pterocarpoides* as a wound healing and anthelmintic agent, giving scientific credence to its use in traditional medicine.

## Highlights

• *Amphimas pterocarpoides* leaves and stem bark are used in traditional medicine.
• The leaf and stem bark ointments increased the rate of wound healing in rats.
• The leaf and stem bark methanol extracts caused worm paralysis and death.
• The leaf and stem bark extracts had high phenolic and flavonoid content.
• Remarkable antioxidant activity was shown by the leaf and bark extracts.

## 1 Introduction

Leguminosae (also called Fabaceae) is the third largest family in the plant kingdom, comprising of about 700 genera with over 19,000 species of trees, shrubs, lianas and herbs which grow in the terrestrial environments of African countries [1]. Plants of the family Leguminosae are well known for their economic use in timber trade and more emphatically for their valuable use in traditional medicine [2]. Several researchers have investigated a number of Leguminosae plants and reported remarkable biological activities including anti-nociception, anti-inflammatory, antioxidant, antimicrobial, anthelmintic, anti-cancer, anti-parasitic effects, indicating the family as a rich source of diversified natural compounds for drug discovery [1]. A wide variety of plant secondary metabolites with great structural diversity including terpenes, alkaloids, polyphenols, cyanogens, polyketides and peptides have been identified in the family Leguminosae with polyphenols including phenolic acids, flavonoids, coumarins and tannins being the most predominant [3, 4, 5, 6].

The genus *Amphimas* a small genus of the Leguminosae family, belonging to the subfamily *Papilionaceae* and confined to West and Central Africa. *Amphimas pterocarpoides* is the most common species of the genus widely distributed throughout tropical African countries including Guinea, DR Congo, Sudan, Gabon, Cameroon, Sierra Leone and Ghana [7]. In Ghana the tree is called ‘*yaya’* and used in traditional medicine [8]. Traditional medicinal uses of the leaves and bark include treatment of cough, pneumonia, venereal diseases, small pox, measles, chicken pox, swellings, pain, wounds, malaria fever and gouty arthritis [9]. A reddish resin exudate from the bark is used in the treatment of dysentery, anaemia, schistosomiasis, haematuria, dysmenorrhea, mumps, and as antidote to several poisons. The twig is applied to prevent miscarriage and a wood decoction is drunk to treat impotence [7]. In phytochemical studies, isoflavonoids and phenolic acids were identified in the roots and stem bark [10, 11, 12]. In previous bioactivity studies, the bark extracts improved mineral density and strength in the bones of oestrogenic deficient rats [13]. Isoflavonoid derivatives including amphiisoflavone, 8-methoxyisoformononetin, 6-methoxyisoformononetin and isoformononetin from the bark and roots demonstrated antimicrobial, antioxidant and oestrogenic activities [14, 15]. The stem bark methanol extract was proven to have a wide safety margin for traditional use [16]. We recently demonstrated that the bark and leaves also possess remarkable anti-inflammatory and antipyretic activities [17]. It is evidenced from literature search that only few scientific reports evaluating the basis of traditional uses of *A. pterocarpoides* exist. The present study was thus intended to further investigate some biological activities of *A. pterocarpoides* bark and leaves including wound healing, anthelmintic and antioxidant activities. HPLC-UV fingerprints were also developed for authentication and quality control.

## 2 Materials and methods

### 2.1 Harvesting and processing of plant material

Fresh samples of the stem bark and leaves of *A. pterocarpoides* were harvested from the Crop Research Institute of Ghana, Fumesua, Ghana [−06°42′51.3″N, 1° 31′44.2″W] in November, 2020. The identity of the samples was confirmed by Dr. George Henry Sam, a botanist at the Herbal Medicine Department, KNUST, Ghana. Herbarium samples with identity codes KNUST/HM1/2018/AP/037 for the leaf and KNUST/HM1/2018/AP/007 for the bark were placed at the Faculty of Pharmacy and Pharmaceutical Sciences Herbarium, KNUST.

### 2.2 Plant extract preparation

The plant material was rid of all extraneous materials by washing with water, cut into smaller chunks, dried in the sun under a shade for 7 days and crushed into coarse powder with a hammer mill (Lab mill machine, Christy and Norris, Chelmsford, England). For the evaluation of wound healing and anthelmintic activities, 600 g each of the powdered stem bark and leaves were extracted separately with 1000 mL of MeOH by cold maceration for 72 h with occasional stirring. The filtrates obtained were concentrated to dried extracts using a rotary evaporator (Rotavapor BUCHI-200, Hamburg Germany) at 50 °C. A dark green semi solid extract for the leaves and a dark brown powdery extract for the stem bark were obtained. The extracts were referred to as APL and APS for the leaf and bark respectively.

For the assessment of antioxidant activity, total phenolic and total flavonoid contents, three solvent extracts were prepared each for the bark and leaves as follows: 1 kg of the powdered sample was extracted with a Soxhlet apparatus successively with 2L each of petroleum ether (pet-ether), ethyl acetate (EtOAc) and methanol (MeOH) in increasing polarity. The extracts obtained were subsequently referred to as APL_P_, APL_E_, APL_M_ and APS_P_, APS_E_, APS_M_ for the pet-ether, EtOAc and MeOH extracts of the bark and leaves respectively.

### 2.3 Qualitative phytochemical screening

Preliminary screening of the powdered samples was undertaken to identify the presence of major classes of secondary metabolites including tannins, glycosides, alkaloids, phytosterols following established methods [18].

### 2.4 Ethical consideration

Ethical approval for the use of experimental animals was obtained from the Animal Ethical Committee (FPPS-AEC/CA01/13), Faculty of Pharmacy and Pharmaceutical Sciences, KNUST. The experimental animals were handled according to the Guidelines for Care and Use of Laboratory Animals (Directive 2010/63/EU; Animal Care and Use Committee, 1998).

### 2.5 Evaluation of wound healing activity

#### 2.5.1 Experimental animals

Sprague-Dawley rats weighing between 90- 130 g were purchased from the Noguchi Memorial Institute for Medical Research, University of Ghana, Accra, Ghana. The animals were housed in the *vivarium* of the Pharmacology Department, KNUST in steel cages (57 × 34 × 40 cm^3^) and fed with commercial rat feed, water *ad libitum*. The following conditions were maintained at the laboratory: temperature: 25 ± 1 °C, relative humidity: 60–70%, 12-h light-dark cycle These conditions were maintained throughout the days of experimental.

#### 2.5.2 Ointment preparation

The leaf (APL) and stem bark (APS) ointments of concentrations 5, 10 and 15% weighing 25 g each were prepared using a scaled formula for simple ointment according to the British Pharmacopoeia [19] (Table 1). Petroleum jelly was used as the base ointment.

**Table 1:** Formula for preparing the aqueous cream with the extract.

Ingredients	Quantities of ingredients (g)		
Base (Petroleum Jelly)	23.7	22.5	21.25
Active ingredient (APL/APS)	1.25	2..5	3.75
Ointment (%)	5.00	10.00	15.00

#### 2.5.3 Evaluation of acute dermal toxicity

Following the guidelines of the Organization for Economic Co-operation and Development (OECD) no. 402, the acute dermal toxicity of the formulated ointments was assessed. This was done to ensure that the ointments were not irritant to the animals' skin at the highest concentrations used. APL (15 %) and APS (15 %) were topically applied on the rats’ bare skin and observed for 14 days [20].

#### 2.5.4 Wound healing activity

The dermal excision wound model in rats was used to assess the wound healing activity of APL and APS ointments [21]. The animals were sedated by hypodermal injection with ketamine (120 mg/kg body weight). The fur at their backs was removed by shaving with a razor blade to achieve a diameter of approximately 4cm. The estimated wound area was drawn on the shaved skin and cleansed with alcohol (70 %). With the aid of surgical blades, toothed forceps and pointed scissors, circular wounds of diameter 2 cm (area = 2.5–3.5 mm^2^) were created. The animals were randomly selected and put into eight groups of five rats each. Treatment of wound started one day post wound creation. The treatment regimen for the groups of rats was as follows: groups 1–3: APL 5, 10 and 15 %; groups 4–6: APS 5, 10 and 15%; group 7 (negative control): normal saline; group 8 (positive control): 1% silver sulphadiazine cream. The open wounds were cleaned with normal saline and treated by topical application of test samples once daily for 21 days.

#### 2.5.5 Measurement of wound contraction

The wound surface area (cm^2^) was measured every other day commencing from day 1 until the 21^st^ day. The wound diameter was taken with digital calipers and the wound surface area was determined by the formula:$wound\;surface\;area\;\left(WSA\right)=\;4\pi r^{2}$; where ***r*** is the radius of the wound**.**

The percentage wound surface closure was obtained from the equation [EQ1]:

[EQ1] $$\%\;wound\;surface\;closure=\frac{WSA\;\left(day\;1\right)-\;WSA\;\left[day\;n\right]}{WSA\;\left(day\;1\right)}\;x\;100$$

Where ‘*n’* represents the day of treatment.

### 2.6 Antioxidant activity

#### 2.6.1 2, 2-diphenyl-1-picrylhydrazyl (DPPH) free radical scavenging assay

The radical scavenging activity was determined according to an established method [22]. The test samples were *A. pterocarpoides* extracts/fractions (31.25–1000 *μ*g/mL), blank MeOH (negative control) and gallic acid (3.125–100 *μ*g/mL, positive control). The experiments were executed in triplicate and the results determined as the mean of three values. The percentage radical scavenging activity (RSA) was determined according to the equation [EQ2]:

[EQ2] $$\%\;Radical\;scavenging\;activity=\frac{Abs\;control-\;Abs\;sample}{Abs\;control}\;x\;100$$

Where ‘Abs sample’ represents the absorbance of test sample/positive control and ‘Abs control’ represents the absorbance of the negative control.

#### 2.6.2 Total antioxidant capacity (TAC)

The antioxidant capacity of the extracts was evaluated following an established method [23]. A line graph [EQ3**:** y = 0.0005850x + 0.07264, R^2^ = 0.8098] of absorbance versus the respective concentration of gallic acid (3.125–100 *μ*g/mL) was obtained. The TAC was deduced from this standard graph and recorded as gallic acid equivalent (GAE) in mg/g of dried extract.

#### 2.6.3 Total phenol content (TPC)

The total phenolic content was evaluated by the Folin-Ciocalteu method [24]. Gallic acid (3.125–100 *μ*g/mL) was used as the reference substance. TPC of extracts was deduced from the line equation, y = 0.003190x + 0.1358, R^2^ = 0.9533 [EQ4], obtained from a graph of absorbance versus the respective concentration of gallic acid (3.125–100 *μ*g/mL). TPC was expressed as gallic acid equivalent (GAE) in mg/g of dried extract.

#### 2.6.4 Total flavonoid content (TFC)

The total flavonoids was quantified by the aluminum chloride colorimetry method [25]. Quercetin (3.125–100 *μ*g/mL) was used as positive control. A line graph [EQ5: y = 0.002382x + 0.05557; (R^2^ = 0.9920) of absorbance versus the respective concentration of quercetin was obtained and the TFC was deduced from this standard graph. TFC was thus recorded as quercetin equivalent (QCE) in mg/g of dried extract.

### 2.7 Anthelmintic activity

#### 2.7.1 Collection of adult *Pheretima posthuma*

The adult Indian earthworm (*Pheretima posthuma*; Order- Haplotaxida) was used for this assay. This worm was selected because of its availability and more importantly, its physiological and anatomical similarity with *Ascaris lumbricoides* (intestinal roundworm) [26]. The earthworms of about 8–10 cm in length and 0.20–0.30 cm in width were collected from the water-logged areas from soil on the banks of Wiwi River in the Botanical Garden at KNUST, Ghana. The worms were washed with 0.9% w/v of normal saline to rid them of dirt and other extraneous matter.

#### 2.7.2 In vitro anthelminthic activity

The anthelmintic activity of the MeOH extracts of the leaves and stem bark of *A. pterocarpoides* was carried out according to a previously described method [27]. The anthelmintic activity of plant extracts was studied by evaluating their effect on worms after direct exposure for a period of time. Different working concentrations of the MeOH extracts was prepared by serial dilution with normal saline to obtain concentrations between 50 and 1.56 mg/mL. The reference drug used was albendazole (10 mg/ml) and normal saline served as the negative control. Briefly, four earthworms each were placed in separate petri dishes filled with 30ml of the extract/normal saline/albendazole. The worms were observed for their natural movements as well as evoked responses. The time taken for the worms to be paralyzed and/or die was recorded. Paralysis was recorded when no movements of any sort (wiggling movement) could be observed except when the worm was shaken vigorously or pricked with a pin. Death of worm was recorded when worms failed to move after being shaken vigorously or when immersed in warm water (50 °C) followed by fading of their skin colour. The results were expressed in comparison to the standard drug albendazole. Concentrations of extracts that produced activity after 200 min were considered inactive.

### 2.8 HPLC fingerprinting

The HPLC fingerprinting of the MeOH extracts (1 mg/mL) was performed for authentication and quality control. The system consisted of a PerkinElmer Flexar HPLC attached to a PDA detector. Separation was achieved on Agilent Zorbax 300SB, C18 (250 × 4.6mm, 5μm) column. The mobile phase consisted of 0.05% Trifluoroacetic acid (A) and methanol (B). A simple linear gradient program beginning with 90% A to 10% B for 3 min, followed by an isocratic step of 90% B for 25 min then a return to the initial 90% A to 10% A for 5 min for re-equilibration of the system between individual runs was used. An injection volume of 20μL, flow rate of 1 ml/min, and a wavelength of 275nm were employed.

### 2.9 Data analysis

Results were recorded as the mean ± standard error of the mean (SEM). Significant differences between the treatment groups and the negative control were determined by the One-Way Analysis of Variance (ANOVA) followed by Dunnet's Multiple Comparisons Test. *P* ≤ 0.05 was considered statistically significant. GraphPad for Windows version 6 (GraphPad Prism Software, San Diego, USA) was used for the analysis.

## 3 Results

### 3.1 Phytochemical analysis

Results of the qualitative phytochemical analysis of the powdered leaf and bark of *A. pterocarpoides* are presented on Table 2.

**Table 2:** Phytochemical screening of leaf and stem bark of *A. pterocarpoides*.

Constituent	Test	Leaf	Stem bark
Tannins	Ferric chloride test	**+**	**+**
Flavonoids	Alkaline reagent test	**+**	**+**
Alkaloids	Dragendorff's test	**-**	**+**
Coumarins	Fluorescence test	**+**	**+**
Glycosides (general)	Fehling's test	**+**	**+**
Saponins	Frothing test	**+**	**+**
Anthraquinones	Borntrager's test	**-**	**-**
Triterpenoids	Salkowski's test	**+**	**+**
Phytosterols	Liebermann Buchard's test	**+**	**+**

(+): Detected; (-): Not Detected.

### 3.2 Wound healing activity

The highest dose of the leaf and bark ointments of *A. pterocarpoides* caused no skin irritation, physical changes or any form of toxic effect to the skin in acute dermal toxicity studies.

**Figure 1:**
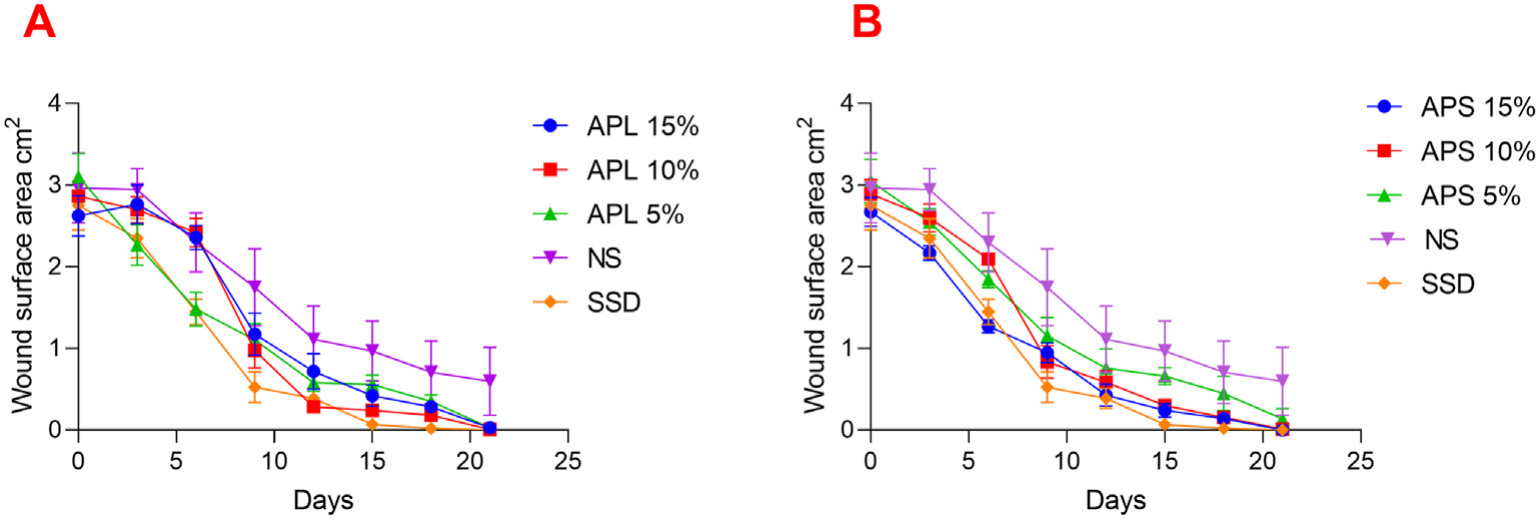
Wound surface area (mm^2^) measured over 21 days of treatment with MeOH extracts of the leaves (**A**) and stem bark (**B**) of *A. pterocarpoides* [APL- *A. pterocarpoides* MeOH leaf extract, APS- *A. pterocarpoides* MeOH stem bark extract, SSD-silver sulphadiazine 1%, NS-normal saline].

Significant (*p* < 0.05) decrease in wound surface area was observed for all extracts (APL and APS) and 1% silver sulphadiazine (SSD)- treated groups compared to the negative control group. Figures 1A and 1B show the gradual reduction in the wound surface diameter estimated every other day beginning from the 3^rd^ day after wound creation. Table 3 records the percentage wound surface closures calculated for all treatment and negative control groups from day 3–21. The stem bark ointment (APS) showed a concentration-dependent wound healing activity while the effect of the leaf ointment (APL) was not concentration-dependent. The overall wound healing potential for test samples was as follows: SSD > APS15% > APS 10% = APL 5% > APL10% > APS 5% = APL 15%. For all extract treated groups, remarkable wound healing (>70% wound surface closure) occurred from day 12–21 whereas the positive control (SSD 1%) exhibited more than 80% wound contraction by day 9 post wound excision. By day 15, significant (*p* < 0.005) wound healing (>90 % wound closure) was observed for 1% SSD, APS 15% and APL 10% treated groups (Figure 1A, Table 3). During the experiment, the rate of wound contraction in the negative control group was remarkably lesser than that of the extracts and SSD-treated groups.

**Table 3:** Wound healing activity of *A. pterocarpoides* leaf and stem bark extracts.

Treatment Group	Percentage (%) wound closure						
	Day 3	Day 6	Day 9	Day 12	Day 15	Day 18	Day 21
APL 5	26.90 ± 6.90	52.23 ± 4.41	64.45 ± 9.54	81.30 ± 5.86	81.96 ± 7.55	88.67 ± 5.05	99.06 ± 4.17 ^*b*^
APL 10	5.89 ± 1.09	15.64 ± 4.74	65.93 ± 5.47	90.03 ± 2.01	91.56 ± 3.85	93.67 ± 3.37	99.68 ± 6.16 ^*b*^
APL 15	5.43 ± 0.57	10.072 ± 4.70	55.17 ± 9.75	72.59 ± 13.49	83.90 ± 12.15	89.19 ± 8.34	95.92 ± 5.88 ^*c*^
APS 5	16.39 ± 2.63	39.36 ± 4.11	62.10 ± 6.61	75.17 ± 6.85	78.32 ± 5.86	85.32 ± 12.1	95.45 ± 6.79 ^*c*^
APS 10	10.14 ± 1.14	27.51 ± 3.38	71.13 ± 6.31	79.78 ± 4.39	89.56 ± 2.83	94.66 ± 4.15	99.59 ± 7.06^*b*^
APS 15	18.87 ± 2.97	52.39 ± 2.85	64.44 ± 6.58	83.93 ± 6.81	90.95 ± 7.85	94.68 ± 7.77	100.00 ^*a*^
SSD 1%	14.62 ± 1.63	47.39 ± 2.01	80.91 ± 6.77	85.91 ± 5.46	97.47 ± 10.59	100.00	100.00^*a*^
NS	0.61 ± 0.06	22.52 ± 3.03	41.06 ± 2.64	62.45 ± 1.60	67.37 ± 2.89	76.11 ± 6.77	79.82 ± 6.94

APL: *A. pterocarpoides* MeOH leaf extract; APS: *A. pterocarpoides* MeOH stem bark extract; NS: normal saline; SSD: silver sulphadiazine; [^*a*^: *p* < 0.005; ^*b*^: *p* < 0.01; ^*c*^: *p* < 0.05- compared to the negative control].

### 3.3 Antioxidant activity

#### 3.3.1 DPPH free radical scavenging activity

**Figure 2:**
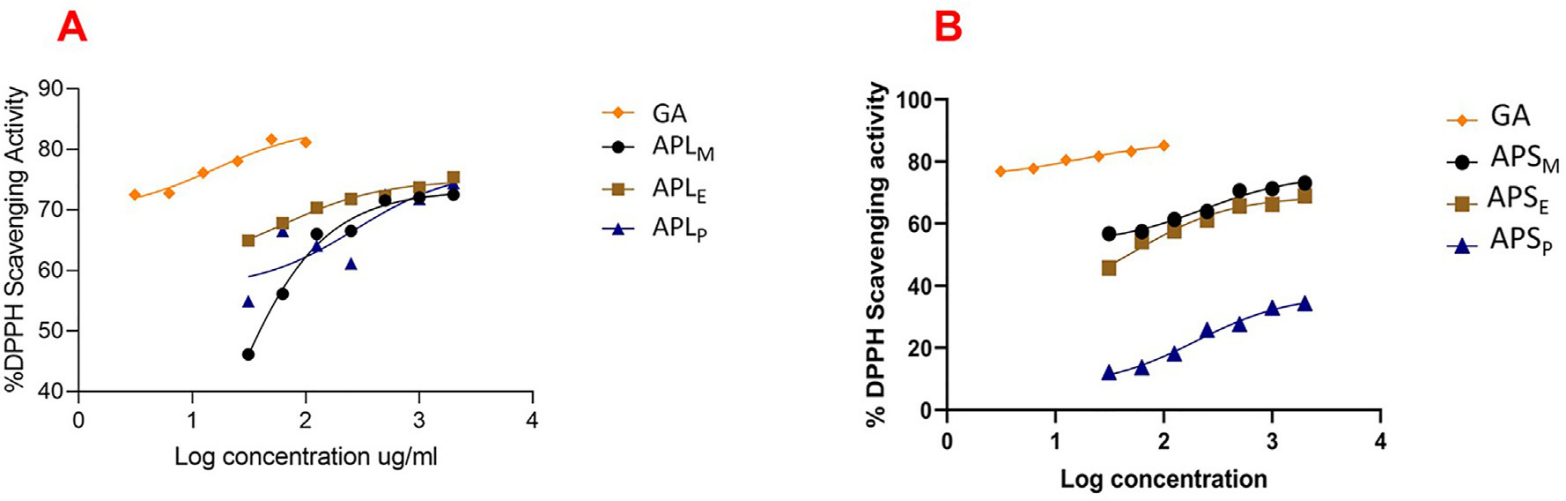
DPPH free radical scavenging of various extracts from the leaves (**A**) and stem bark (**B**) of *A. pterocarpoides* [APL_M_- *A. pterocarpoides* MeOH leaf extract, APL_E_- *A. pterocarpoides* EtOAc leaf extract; APL_P_- *A. pterocarpoides* pet-ether leaf extract; APS_M_- *A. pterocarpoides* MeOH stem bark extract; APS_E_- *A. pterocarpoides* EtOH stem bark extract, APS_P_- *A. pterocarpoides* pet-ether stem bark extract, GA-gallic acid].

The DPPH free radical scavenging activity of the various solvent extracts of the leaf and stem bark of *A. pterocarpoides* was expressed as IC_50_s obtained from non-linear regression curves as shown in Figure 2. From the results, both bark and leaf extracts displayed remarkable free radical scavenging effect which increased with increasing concentration. In general, the stem bark extracts (IC_50_ range from 15.38 to 199.20 μg/mL) had a higher radical scavenging effect than the leaf extracts (IC_50_ range from 23.30 to 278.4 μg/mL). The MeOH extracts of the bark and leaves had the highest radical scavenging effect with IC_50_s of 15.38 and 23.30 μg/mL respectively comparable to that of gallic acid (IC_50_ = 15.62 μg/mL). The radical scavenging effect of test extracts was as follows: APS_M_ > Gallic acid > APL_M_ > APS_E_ > APL_E_ > APS_P_ > APL_P_. The IC_50_s of all samples including the positive control are presented on Table 4.

**Table 4:** Antioxidant activity of the leaf and stem bark of *A. pterocarpoides*.

	DPPH (IC_50_ (μg/mL)	TAC (mg/g GAE)	TPC (mg/g GAE)	TFC (mg/g QCE)
APL_M_	23.30	124.6 ± 1.00	84.54 ± 1.36	130.7 ± 1.71
APL_E_	63.14	67.12 ± 0.63	48.47 ± 1.27	54.44 ± 2.37
APL_P_	278.4	57.38 ± 2.10	45.91 ± 0.58	53.19 ± 3.54
APS_M_	15.38	126.0 ±1.06	225.0 ± 20.0	201.0 ± 1.41
APS_E_	56.42	105.0 ± 7.02	82.0 ± 7.07	157.0 ± 2.89
APS_P_	199.20	86.0 ± 1.41	52.7 ± 0.06	82.50 ± 3.56
Gallic acid	15.62	-	-	-

APL_M_- *A. pterocarpoides* MeOH leaf extract, APL_E_- *A. pterocarpoides* EtOAc leaf extract; APL_P_- *A. pterocarpoides* pet-ether leaf extract; APS_M_- *A. pterocarpoides* MeOH stem bark extract; APS_E_- *A. pterocarpoides* EtOH stem bark extract, APS_P_- *A. pterocarpoides* pet-ether stem bark extract, GAE-gallic acid equivalent, QCE-quercetin equivalent.

#### 3.3.2 Total antioxidant capacity (TAC)

**Figure 3:**
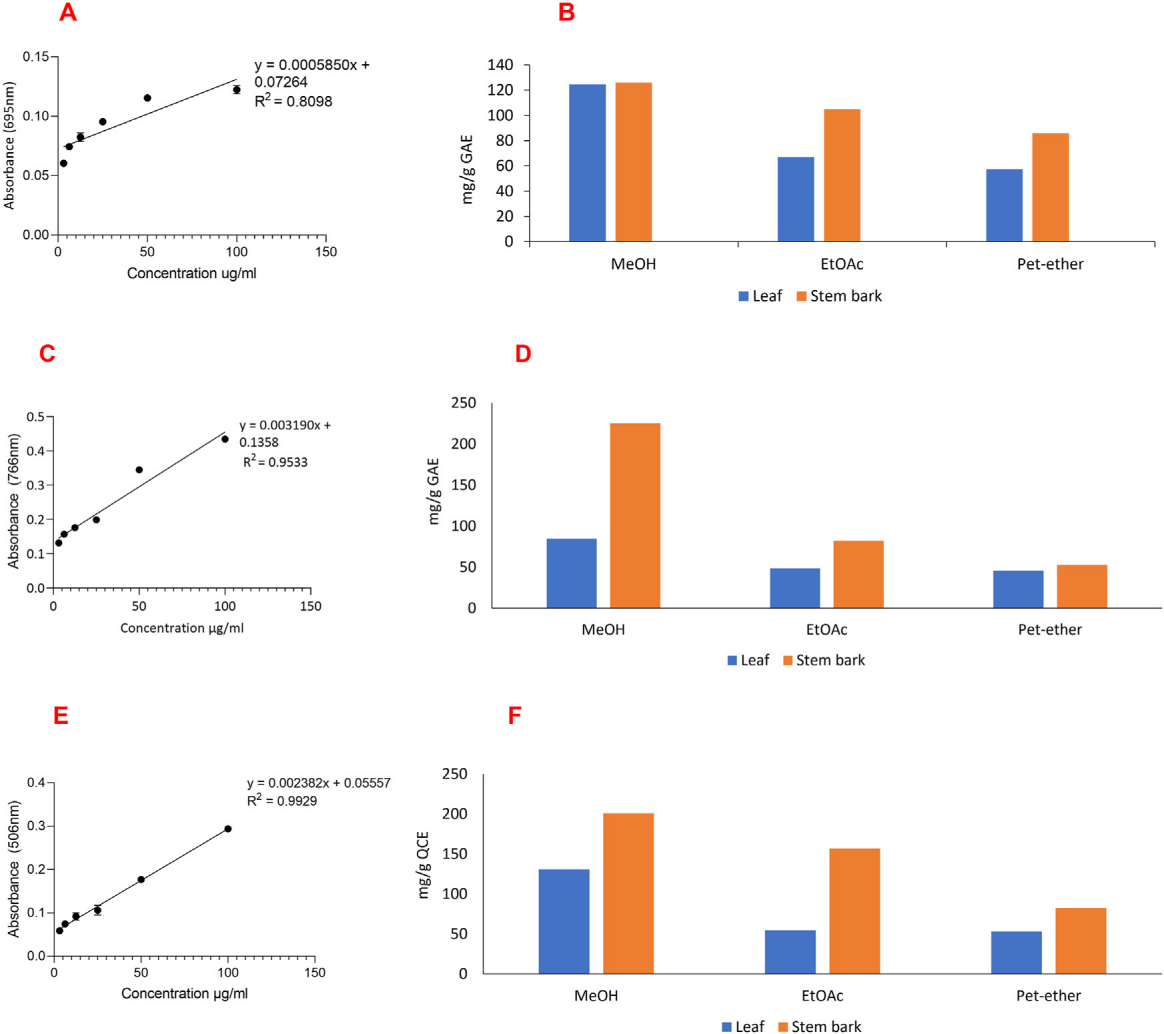
Total antioxidant capacity (**A, B**), total phenolic content (**C, D**) and total flavonoid content (**E, F**) of the leaves and stem bark of the MeOH, EtOAc and pet-ether extracts of *A. pterocarpoides*.

The total antioxidant capacity was determined from a standard line graph [EQ3] developed using gallic acid (Figure 3A) and was thus recorded as gallic acid equivalent (GAE). Significant variations were observed among the TAC of the leaf and bark extracts which also varied according to the type of solvent extract (Table 4). For both leaf and bark, TAC increased in the order APS_M_ > APL_M_ > APS_E_ > APS_P_ > APL_E_ > APL_P_ (Figure 3B). The MeOH extracts had the highest TAC. Generally, the stem bark extracts had a higher TAC than the leaf extracts. Comparing the TAC of the various solvent extracts, it was observed that no significant difference existed between the TAC of the MeOH extracts of the leaves and stem bark (APS_M_ = 126. 0 ± 1.06; APL_M_ 124.6 ± 1.00 mg/g GAE). However, the EtOAc extracts of the bark and leaves had higher TAC than the pet-ether extracts (Table 4, Figure 3B).

#### 3.3.3 Total phenolic content (TPC)

The total phenolic content in the leaves and stem bark of *A. pterocarpoides* was determined from a standard curve of gallic acid [EQ4] (Figure 3C) and was expressed as gallic acid equivalent (GAE). The total phenolic content varied between the two plant parts and also with respect to the solvent used for extraction. The stem bark had a higher phenolic content (52.7–225.0 mg/g GAE) than the leaves (45.91–84.54 mg/g GAE). Over all the MeOH extracts of the stem bark (225.0 ± 20.08 mg/g GAE) and leaves (84.54 ± 1.36 mg/g GAE) recovered the highest amount of phenolic content compared to other solvent extracts (Figure 3D). TPC decreased with decreasing polarity of solvent hence the pet-ether fraction has the least TPC. In the leaf extract there was no significant difference between the TPC of the EtOAc and pet-ether extracts (Table 4).

#### 3.3.4 Total flavonoid content (TFC)

The total flavonoid content was determined from the standard curve of quercetin [EQ5] (Figure 3E) and was expressed as quercetin equivalent (QCE). The TFC for the leaf extracts ranged from 53.19 to 130.7 mg/g QCE and from 82.50 to 201.0 mg/g QCE for the stem bark (Table 4). Among the three solvents used, methanol extracted the maximum amounts of flavonoids, followed by ethyl acetate. Pet-ether was the least effective in extracting flavonoids from both leaves and stem bark. The stem bark had a higher flavonoid content (201.0 ± 1.41 mg/g QCE) than the leaf extract (130.7 ± 1.71 mg/g QCE). There was no significant difference between the TFC of EtOAc and pet-ether fractions of the leaf (Table 4; Figure 3F).

### 3.4 Anthelmintic activity

The anthelmintic activity of the crude MeOH leaf (APL) and the stem bark (APS) extracts were recorded as paralysis time and death time in minutes. The results are presented on Table 5.

**Table 5:** Anthelmintic activity of the leaf and stem bark of *A. pterocarpoides*.

Treatment	Concentration (mg/mL)	Time of paralysis (min) (Mean ± SEM)	Time of death (min) (Mean ± SEM)
Normal saline	-	NP	ND
Albendazole	10	76.30 ± 0.721	117.4 ± 1.209
APL	50	24.90 ± 0.781	34.60 ± 1.127
	25	43.80 ± 1.212	54.00 ± 1.670
	12.5	59.73 ± 1.289	70.30 ± 0.600
	6.25	82.60 ± 0.300	93.27 ± 0.723
	3.12	110.57 ± 0.208	151.10 ± 3.538
	1.56	163.80 ± 0.264	200.73 ± 1.331
APS	50	27.23 ± 0.711	54.87 ± 1.500
	25	47.97 ± 1.900	69.87 ± 1.650
	12.5	75.70 ± 1.450	93.27 ± 1.193
	6.25	180.04 ± 1.264	130.03 ± 8.328
	3.12	130.00 ± 0.702	175.50 ± 0.360
	1.56	163. 23 ± 0.305	ND

APL: *A. pterocarpoides* MeOH leaf extract; APS: *A. pterocarpoides* MeOH stem bark extract; NP: no paralysis; ND: no death.

### 3.5 HPLC fingerprinting

**Figure 4:**
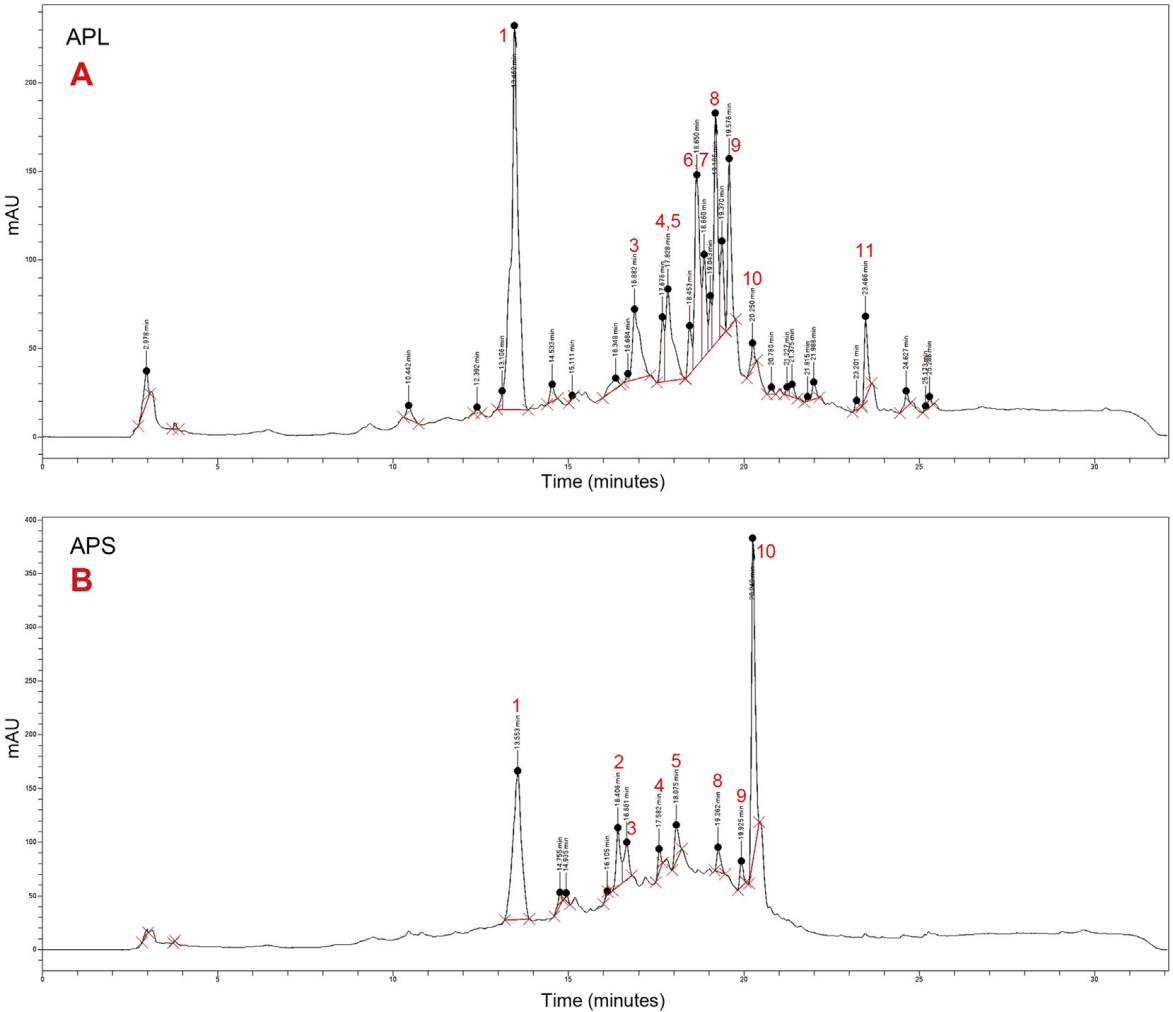
HPLC chromatograms of the MeOH extracts of the leaves (**A**) and stem bark (**B**) of *A. pterocarpoides* (measured at 275 nm).

The chemical fingerprints of the MeOH leaf (APL) and stem bark (APS) extracts (1 mg/mL) were determined for the purpose of authentication and quality control (Figure 4). Due to potential peak shifting, which could arise from variations in chromatographic conditions, the retention times were converted to relative retention times. Briefly, one prominent peak was selected as the reference peak to calculate the relative retention times of other peaks in each chromatogram. From the results, ten prominent peaks were identified in the leaf extract and eight in the stem bark extract (Table 6). By comparison of their relative retention times, seven peaks were common to both leaf and stem bark extracts. For all peaks (except peak 10), higher intensities were observed in the leaf extract than in the stem bark extract indicating relatively higher amounts of the individual components in the leaf.

**Table 6:** Retention and relative retention times for major peaks in the MeOH extracts of the leaf and stem bark of *A. pterocarpoides*.

Peak	APL		APS	
	Retention time (min)	Relative retention time (min)	Retention time (min)	Relative retention time (min)
1.	13.46	1.00	13.55	1.00
2.	-	-	16.41	0.83
3.	16.88	0.80	16.66	0.80
4.	17.68	0.77	17.58	0.77
5.	17.83	0.75	18.08	0.75
6.	18.65	0.72	-	-
7.	18.86	0.71	-	-
8.	19.19	0.70	19.26	0.70
9.	19.58	0.68	19.93	0.68
10.	20.25	0.66	20.25	0.66
11.	23.47	0.57	-	-

APL: *A. pterocarpoides* MeOH leaf extract; APS: *A. pterocarpoides* MeOH stem bark extract.

## 4 Discussion

The present investigation describes the wound healing and anthelmintic activity of the crude methanol extracts of the stem bark and leaves of *A. pterocapoides* as well as a comparative evaluation of the antioxidant activity, phenolic and flavonoid contents of various solvent fractions from both plant parts. HPLC fingerprints of the extracts were also developed for quality control.

Qualitative phytochemical screening of the powdered leaves and bark showed the presence of saponins, triterpenoids, flavonoids, phytosterols, tannins and coumarins in both plant parts. Alkaloids were detected only in the bark. For the quality control of herbal extracts, several authors have utilized chromatography coupled with spectral techniques such as HPLC-UV and HPLC-MS to identify and quantify marker compounds in extracts [28, 29]. For most herbal extracts however, the complexity of mixtures of phytoconstituents makes identifying potential marker compounds usually difficult. Sometimes, the active principles have not been conclusively identified and can thus not be accurately quantified. Some studies have recommended the evaluation of whole extracts by a process called chemical fingerprinting where the chromatographic pattern of chemically characteristic constituents in the extract is developed [30]. By the concept of phytoequivalence, the chromatographic fingerprint of a plant extract measured under the same conditions will consistently demonstrate the exact similarity and/or differences and can thus be used for authentication and quality control of specific species [31]. In this study, HPLC fingerprints have been developed for the MeOH extracts of *A. pterocarpoides* leaves and stem bark. Together with other parameters [17], these chromatograms can be used for authentication and quality assessment of *A. pterocarpoides* crude samples and herbal preparations.

The methanol extracts of the bark and leaves *of A. pterocarpoides* displayed significant increase in the rate of wound contraction, indicating a clear response to treatment unlike the negative control group. Plant secondary metabolites such as tannins, flavonoids and triterpenoids are known to positively influence wound healing [32]. The MeOH extracts were also found to contain high amounts of phenolic compounds and flavonoids. Tchoumtchoua et al., (2013) identified several isoflavonoid derivatives in the stem bark of *A. pterocarpoides* [10]. Flavonoids are known to significantly influence wound healing by their anti-inflammation, antioxidant and angiogenesis properties which promote wound contraction, capillary regeneration, collagen deposition and re-epithelialization [33]. Tannins are able to stimulate dermal fibroblasts and keratinocytes, resulting in an increased secretion of collagen and regeneration of epithelial cells [34]. Triterpenes reduce inflammation and the generation of reactive oxygen species in the wound microenvironment, expediting the tissue repair process [35, 36]. These phytoconstituents also have antimicrobial and astringent properties which promote wound healing as well as improve the rate of epithelialization [37]. In our previous study, the stem bark and leaves of *A. pterocarpoides* demonstrated remarkable anti-inflammatory activity which supports the observed wound healing effect [17]. The wound healing activity of *A. pterocarpoides* could thus be attributed to the individual or combined effect of the constituents in the plant.

The role of antioxidants in enhancing and accelerating wound healing cannot be underestimated [38]. Among plant secondary metabolites, phenolic compounds such as phenolic acids, tannins, flavonoids and coumarins have shown promising antioxidant effects in several models [32]. This study has revealed that the MeOH extracts of the bark and leaves contained the highest amounts of phenolic constituents and flavonoids compared to the EtOAc and pet-ether fractions. The results was congruent with previous studies which also reported MeOH as the best solvent for extracting phenolic compounds [39, 40]. Tchoumtchoa et al., (2013) recommended the use of methanol as solvent for extracting maximum amount of phenolic content from *A. pterocarpoides* [10]. Polar solvents are the most suitable for maximum extraction of phenolic compounds due to the chemical nature of the latter. Remarkable DPPH radical scavenging and total antioxidant capacity was also exhibited by the MeOH fractions of both stem bark and leaf. In a previous study, the MeOH–CH_2_Cl_2_ (1:1) extract of *A. petrocarpoides* roots exhibited DPPH radical scavenging effect (IC_50_ = 63.59) [14]. Previous works have demonstrated a positive correlation between phenolic content, antioxidant activity and wound healing effects of plant extracts [41, 42]. This suggests that the amount of phenolic content in *A. pterocarpoides* greatly influences its antioxidant activity and in turn its wound healing activity.

The stem bark of *A. pterocarpoides* is used in the treatment of schistosomiasis in traditional medicine, giving an indication of possible effect on helminth parasites. The stem bark and leaf MeOH extracts demonstrated a concentration- and time-dependent paralysis and mortality of the adult earth worm, *P. posthuma*. Overall, the leaf extract showed a better anthelmintic effect than the stem bark extract. At an exposure concentration of 6.25 mg/mL, APL caused worm paralysis at 82.60 min almost comparable to the effect of the standard anthelmintic drug, albendazole (10 mg/mL) which gave paralysis at 76.30 min. Interestingly, APL (6.25 mg/mL) caused death of worms at the 93^rd^ minute better than albendazole (10 mg/mL) which caused death at 117^th^ minute. The anthelmintic effect of medicinal plants has been attributed to constituents such as terpenoids, alkaloids and polyphenols particularly tannins [43, 44]. Tannins, have been shown to possess significant anthelmintic activity against a wide range of nematodes through interactions with proline-rich proteins on the worm cuticle or gastrointestinal tract interfering with worm motility, feeding and absorption of nutrients in the intestines [45]. Saponins are generally known to interact with cell membrane causing changes in membrane permeability resulting in possible alteration of some biological functions [26, 46]. Alkaloids may affect the central nervous system resulting in paralysis and death of worms [43]. The occurrence of these phytochemicals in the leaves and bark of *A. pterocarpides* may influence its anthelmintic effect.

The current results provide first-hand information on some bioactivities of the bark and leaves of *A. pterocarpoides*. Further investigations on the exact mechanisms of action as well as phytochemical analysis employing chromatography and spectrophotometric methods such as mass spectrometry to identify specific compounds responsible for the observed bioactivities are imperative and underway.

## 5 Conclusion

This study has provided scientific evidence that topical application of *A. pterocarpoides* leaves and stem bark ointments on skin lesions accelerates wound healing. The MeOH extracts of the leaf and stem bark also possess remarkable anthelmintic and antioxidant activity attributable to appreciable amounts of phenolic and flavonoid content. HPLC chromatograms developed for the leaves and stem bark can serve as fingerprints for the quality assessment of preparations containing *A. pterocarpoides* leaf and stem bark. The presence tannins, flavonoids, saponins, coumarins, alkaloids and triterpenoids in both plant parts may be responsible for the observed therapeutic effects of the plant in traditional medicine.

## Declarations

### Author contribution statement

Evelyn Asante-Kwatia: Conceived and designed the experiments; Performed the experiments; Analyzed and interpreted the data; Contributed reagents, materials, analysis tools or data; Wrote the paper.

Silas Adjei: Performed the experiments; Analyzed and interpreted the data; Wrote the paper.

Yakubu Jibira: Performed the experiments; Analyzed and interpreted the data; Contributed reagents, materials, analysis tools or data; Wrote the paper.

Lord Gyimah: Performed the experiments; Analyzed and interpreted the data.

George Adjei-Hinneh: Contributed reagents, materials, analysis tools or data.

Isaac Kingsley Amponsah: Conceived and designed the experiments; Contributed reagents, materials, analysis tools or data.

Abraham Yeboah Mensah: Conceived and designed the experiments.

### Funding statement

This research did not receive any specific grant from funding agencies in the public, commercial, or not-for-profit sectors.

### Data availability statement

Data included in article/supplementary material/referenced in article.

### Declaration of interests statement

The authors declare no conflict of interest.

### Additional information

No additional information is available for this paper.

## References

[1] Benjamim J.K., da Costa K.A., Santos A.S. (2021). Chemical, botanical and pharmacological aspects of the Leguminosae. Phcog. Rev..

[2] Catarino S., Duarte M.C., Costa E., Carrero P.G., Romeiras M.M. (2019). Conservation and sustainable use of the medicinal Leguminosae plants from Angola. PeerJ.

[3] Singh H., Chahal P., Mishra A., Mishra A.K. (2020). An up-to-date review on chemistry and biological activities of *Senna occidentalis* (L.) Link Family: Leguminosae. Adv. Trad. Med..

[4] Veitch N.C. (2007). Isoflavonoids of the Leguminosae. Nat. Prod. Rep..

[5] Chew Y.L., Chan E.W., Tan P.L., Lim Y.Y., Stanslas J., Goh J.K. (2011). Assessment of phytochemical content, polyphenolic composition, antioxidant and antibacterial activities of Leguminosae medicinal plants in Peninsular Malaysia. BMC Compl. Alternative Med..

[6] Lacaille-Dubois M.A., Pegnyemb D.E., Noté O.P., Mitaine-Offer A.C. (2011). A review of acacic acid-type saponins from Leguminosae-Mimosoideae as potent cytotoxic and apoptosis inducing agents. Phytochemistry Rev..

[7] Tchinda A., Tané P. (2008). Plant Resources of Tropical Africa Timber 1.

[8] Ayarkwa J. (1994). Strength properties of yaya (*Amphimas pterocarpoides*). Ghana J. For..

[9] Burkill H.M. (1994).

[10] Tchoumtchoua J., Njamen D., Mbanya J.C., Skaltsounis A.L., Halabalaki M. (2013). Structure oriented UHPLC-LTQ Orbitrap-based approach as a dereplication strategy for the identification of isoflavonoids from *Amphimas pterocarpoides* crude extract. J. Mass Spectrom..

[11] Bevan C.W., Ekong D.E., Obasi M.E., Powell J.W. (1966). West African timbers. Part XIII. Extracts from the heartwood of *Amphimas pterocarpoides* and *Pterocarpus erinaceous*. J. Chem. Soc. C Org..

[12] Tchoumtchoua J., Halabalaki M., Njamen D., Mbanya J., Skaltsounis L. (2010). Isolation of isoflavonoids from *Amphimas pterocarpoides* and structure elucidation using LC-HRMSn and NMR techniques. Planta Med..

[13] Patsaki A., Tchoumtchoua J., Passali C., Lelovas P., Kourkoulis S. (2016). The protective effect of *Amphimas pterocarpoides* plant extract on bone mineral density and strength in estrogen deficient ovariectomized Wistar Rats. Med. Aromatic Plants.

[14] Saah E.P., Sielinou V.T., Kuete V., Lacmata S.T., Nkengfack A.E. (2013). Antimicrobial and antioxidant isoflavonoid derivatives from the roots of *Amphimas pterocarpoides*. Z. Naturforsch. B Chem. Sci..

[15] Tchoumtchoua J., Makropoulou M., Ateba S.B., Boulaka A., Halabalaki M., Lambrinidis G., Meligova A.K., Mbanya J.C., Mikros E., Skaltsounis A.L., Mitsiou D.J. (2016). Estrogenic activity of isoflavonoids from the stem bark of the tropical tree *Amphimas pterocarpoides*, a source of traditional medicines. J. Steroid Biochem. Mol. Biol..

[16] Tchoumtchoua J., Mouchili O.R., Ateba S.B., Zingue S., Halabalaki M., Mbanya J.C., Skaltsounis A.L., Njamen D. (2014). Safety assessment of the methanol extract of the stem bark of *Amphimas pterocarpoides* harms: acute and subchronic oral toxicity studies in Wistar rats. Toxicol. Rep..

[17] Adjei-Hinneh G., Komlaga G., Asante-Kwatia E., Mensah A.Y. (2021). Quality control standardization and evaluation of the anti-inflammatory and antipyretic effects of the leaves and stem bark of *Amphimas pterocarpoides* harms (Leguminosae). J. Pharmacogn. Phytotherapy.

[18] Evans W.C. (2009).

[19] British Pharmacopoeia (2009).

[20] Kokane D.D., More R.Y., Kale M.B., Nehete M.N., Mehendale P.C., Gadgoli C.H. (2009 Jul 15). Evaluation of wound healing activity of root of *Mimosa pudica*. J. Ethnopharmacol..

[21] Dwivedi D., Dwivedi M., Malviya S., Singh V. (2017). Evaluation of wound healing, anti-microbial and antioxidant potential of *Pongamia pinnata* in wistar rats. J. Tradit. Complement. Med..

[22] Sharma O.P., Bhat T.K. (2009). DPPH antioxidant assay revisited. Food Chem..

[23] Apak R., Gorinstein S., Böhm V., Schaich K.M., Özyürek M., Güçlü K. (2013). Methods of measurement and evaluation of natural antioxidant capacity/activity (IUPAC Technical Report). Pure Appl. Chem..

[24] McDonald S., Prenzler P.D., Antolovich M., Robards K. (2001). Phenolic content and antioxidant activity of olive extracts. Food Chem..

[25] Chang C.C., Yang M.H., Wen H.M., Chern J.C. (2002). Estimation of total flavonoid content in propolis by two complementary colometric methods. J. Food Drug Anal..

[26] Ali N., Shah S.W., Shah I., Ahmed G., Ghias M., Khan I. (2011). Cytotoxic and anthelmintic potential of crude saponins isolated from *Achillea Wilhelmsii* C. Koch and *Teucrium Stocksianum* boiss. BMC Compl. Alternative Med..

[27] Adu F., Agyare C., Sam G.H., Boakye Y.D., Boamah V.E. (2018). Anthelmintic resistance modifying properties of extracts of *Cyperus difformis* L.(Cyperiaceae). Invest. Med. Chem. Pharmacol..

[28] Al-Huqail A.A., Behiry S.I., Salem M.Z., Ali H.M., Siddiqui M.H., Salem A.Z. (2019). Antifungal, antibacterial, and antioxidant activities of *Acacia saligna* (Labill.) HL Wendl. flower extract: HPLC analysis of phenolic and flavonoid compounds. Molecules.

[29] Adjei S., Amponsah I.K., Bekoe S.O., Harley B.K., Mensah K.B., Mensah A.Y., Baah M.K., Fosu-Mensah G. (2021). Fruits of *Vitex doniana* Sweet: toxicity profile, anti-inflammatory and antioxidant activities, and quantification of one of its bioactive constituents oleanolic acid. Heliyon.

[30] Lalit G., Andola H.C., Purohit V.K., Rawat M.S., Rawal R.S., Bhatt I.D. (2010). Chromatographic and spectral fingerprinting standardization of traditional medicines: an overview as modern tools. Res. J. Phytochem..

[31] Leach D., Fryganas C., Mueller-Harvey I., Wohlmuth H. (2017). Phytoequivalence of therapeutic cinnamon barks and extracts with low coumarin levels. Planta Med. Int. Open.

[32] Barku V.Y. (2019).

[33] Carvalho M.T., Araújo-Filho H.G., Barreto A.S., Quintans-Júnior L.J., Quintans J.S., Barreto R.S. (2021). Wound healing properties of flavonoids: a systematic review highlighting the mechanisms of action. Phytomedicine.

[34] Agyare C., Lechtenberg M., Deters A., Petereit F., Hensel A. (2011). Ellagitannins from *Phyllanthus muellerianus* (Kuntze) Exell.: geraniin and furosin stimulate cellular activity, differentiation and collagen synthesis of human skin keratinocytes and dermal fibroblasts. Phytomedicine.

[35] Agra L.C., Ferro J.N., Barbosa F.T., Barreto E. (2015). Triterpenes with healing activity: a systematic review. J. Dermatol. Treat..

[36] Ghiulai R., Roşca O.J., Antal D.S., Mioc M., Mioc A., Racoviceanu R., Macaşoi I., Olariu T., Dehelean C., Creţu O.M., Voicu M. (2020). Tetracyclic and pentacyclic triterpenes with high therapeutic efficiency in wound healing approaches. Molecules.

[37] Shetty S.B. (2013). Wound healing and indigenous drugs: role as antioxidants: a Review. Res. Rev.: J. Med. Health Sci..

[38] Fitzmaurice S.D., Sivamani R.K., Isseroff R.R. (2011). Antioxidant therapies for wound healing: a clinical guide to currently commercially available products. Skin Pharmacol. Physiol..

[39] Ghatak A., Nair S., Vajpayee A., Chaturvedi P., Samant S., Soley K., Kudale S., Desai N. (2015). Evaluation of antioxidant activity, total phenolic content, total flavonoids, and LC-MS characterization of *Saraca asoca* (Roxb.) De. Wilde. Int. J. Adv. Res..

[40] Stankovic M.S. (2011). Total phenolic content, flavonoid concentration and antioxidant activity of Marrubium peregrinum L. extracts. Kragujevac J. Sci..

[41] Agyare C., Dwobeng A.S., Agyepong N., Boakye Y.D., Mensah K.B., Ayande P.G., Adarkwa-Yiadom M. (2013). Antimicrobial, antioxidant, and wound healing properties of *Kigelia africana* (Lam.) Beneth. and *Strophanthus hispidus* DC. Adv. Pharmacol. Sci..

[42] Baidoo M.F., Mensah A.Y., Ossei P.P., Asante-Kwatia E., Amponsah I.K. (2021). Wound healing, antimicrobial and antioxidant properties of the leaf and stem bark of *Entada africana*. Guill. & Perr. South Afr. J. Botany..

[43] Zaman M.A., Abbas R.Z., Qamar W., Qamar M.F., Mehreen U., Shahid Z., Kamran M. (2020). Role of secondary metabolites of medicinal plants against *Ascaridia galli*. World Poultry Sci. J..

[44] Asante-Kwatia E., Mensah A.Y., Gyimah L., Mensah A.D.F.Y. (2021). Pharmacognosy-Medicinal Plants.

[45] Williams A.R., Fryganas C., Ramsay A., Mueller-Harvey I., Thamsborg S.M. (2014). Direct anthelmintic effects of condensed tannins from diverse plant sources against *Ascaris suum*. PLoS One.

[46] Maestrini M., Tava A., Mancini S., Tedesco D., Perrucci S. (2020). In vitro anthelmintic activity of saponins from *Medicago spp*. against sheep gastrointestinal nematodes. Molecules.

